# Follicular Helper T Cell Derived Exosomes Promote B Cell Proliferation and Differentiation in Antibody-Mediated Rejection after Renal Transplantation

**DOI:** 10.1155/2019/6387924

**Published:** 2019-05-15

**Authors:** Jintao Yang, Lili Bi, Xiuyun He, Zhen Wang, Yeyong Qian, Li Xiao, Bingyi Shi

**Affiliations:** ^1^Medical School of Chinese PLA, Chinese PLA General Hospital, Beijing 100853, China; ^2^Beijing Key Laboratory of Immunology Regulatory and Organ Transplantation, Basic Research Lab of Organ Transplant Institute, The Eighth Medical Center of Chinese PLA General Hospital, Beijing 100091, China

## Abstract

Follicular helper T cells (Tfh cells) are closely related to the occurrence and development of antibody-mediated rejection (AMR) after renal transplantation. Exosomes play a key role in the rejection after organ transplantation. However, whether Tfh-derived exosomes are involved in AMR has not been reported. We collected peripheral blood from 42 kidney transplant patients and found no significant differences in CD4+CXCR5+ and CD4+CXCR5+CXCR3+CCR6-exosomes between AMR and non-AMR groups, whereas the proportion of CD4+CXCR5+CXCR3-exosomes was significantly higher in AMR group than that in non-AMR group; CTLA-4 expression of CD4+CXCR5+exosomes was significantly lower in AMR group than that in non-AMR group. HLA-G expression was not significantly different between two groups. We further separated CD4+CXCR5+cells from patients by magnetic beads. Coculture experiments showed that Tfh cell-derived exosomes in AMR patients significantly promoted B cell proliferation and differentiation, compared with non-AMR group, the percentage of B cells and plasma cells increased by 87.52% and 110.2%, respectively. In conclusion, our study found that Tfh cell-derived exosomes could promote the proliferation and differentiation of B cells and they may play an important role in the development of AMR after renal transplantation.

## 1. Introduction

Renal transplantation is the most effective way to treat end-stage renal disease and improve the life quality of dialysis patients. The use of immunosuppressive drugs significantly reduces T cell-mediated rejection after renal transplantation [1]. However, the long-term prognosis of renal transplant patients is not ideal. Antibody-mediated rejection (AMR) has gradually become a major cause of late renal transplantation failure. The treatment of AMR includes plasmapheresis and drug therapy, but these methods have large side effects and poor efficacy [2, 3]. Therefore, it is of great clinical significance to search new therapeutic targets.

Follicular helper T cell (Tfh cell) is one of helper T cell subset and necessary to assist B cells in differentiating into plasma cells. Tfh cells promote B cell proliferation, differentiation, and affinity maturation by direct contact in the germinal center or secretion of IL-21 [4, 5]. Tfh cells are closely related to the occurrence and development of AMR after renal transplantation [6, 7]. Our previous study found that the proportion of IL-21-producing Tfh cell subsets (Tfh2 and Tfh17 cells) was significantly higher in the AMR group than that in non-AMR group [8].

In the extracellular vesicles released by cells, there is a subpopulation of 30-150 nm in diameter that originates in the endocytic pathway, called exosomes. Exosomes can transfer specific components such as nucleic acids, proteins, and lipids between cells and have important functions of mediating cell-to-cell communication [9, 10]. Different cell-derived exosomes have different functions and can serve as biomarkers for disease diagnosis [11–13]. At present, the effect of Tfh cell-derived exosomes on B cell proliferation and differentiation is unclear, and their relationship with AMR has not yet been reported. The study of the relationship between Tfh cell-derived exosomes and AMR not only provides a new perspective for elucidating the mechanism of Tfh cell-assisted B cell proliferation and differentiation but also provides a new approach for the prevention and monitoring of AMR.

## 2. Materials and Method

### 2.1. Collection of Clinical Samples

Our study included patients with end-stage renal disease who had undergone a kidney transplant in The Eighth Medical Center of Chinese PLA General Hospital. Exclusion criteria included cotransplantation, age less than 18 years, severe infection, and stimulation of bone marrow hematopoiesis. This study was approved by the Ethics Committee of The Eighth Medical Center of Chinese PLA General Hospital and was performed according to the principles of the Declaration of Helsinki. The experiments involving human subjects were approved by Institutional Review Board of The Eighth Medical Center of Chinese PLA General Hospital. All participants provided written informed consent, and the ethics committee approved the consent procedure.

### 2.2. Separation of Exosomes

The peripheral blood of control and chronic allograft dysfunction (CRAD) patients (CRAD is further divided into AMR group and non-AMR group) was collected. Serum was extracted from 2 mL peripheral blood through centrifugation, and exosomes were isolated according to the instructions of ExoQuickTM Exosome Precipitation Solution Kit (SBI Corporation) and the detailed steps were as follows. Cell debris was removed by centrifugation at 3000 g for 15 min at room temperature. The supernatant was transferred to a 1.5 ml EP tube. 250 *μ*l of the sample was transferred into a dry 1.5 ml EP tube, 63 *μ*l of ExoQuick™ Exosome Precipitation Solution reagent (SBI Corporation) was added then mixed until the sample was completely dissolved. In the upright state, the mixture tube was placed in a refrigerator at 4°C for 30 minutes, centrifuged at 1500×g for 30 minutes, and the supernatant was discarded. The remaining substance in the tube was exosomes. They were dissolved in 100 *μ*l PBS for further testing. Transmission electron microscopy and nanoparticle tracking analysis (NTA) technique were used to detect the basic characteristics of exosomes.

### 2.3. Exosomal Biomarkers Detection by Flow Cytometry

20 *μ*l of magnetic beads in Exosome-Human CD63 Isolation/Detection Reagent (Invitrogen) were used, washed once with separation buffer, and 100 *μ*l of exosome solution was added and incubated on a sample mixer that tilted and rotated at 4°C for 22 hours. After washing the magnetic beads 3 times with the separation buffer, exosomes were divided into two parts. One part was added with anti-CD63, anti-CD4, anti-CXCR5, anti-CXCR3, and anti-CCR6 antibodies (ALL purchased from BD), and the other part was added with anti-CD63, anti-CD4, anti-CXCR5, anti-HLA-G, and anti-CTLA-4 flow antibodies (ALL purchased from BD); all of them were incubated on the sample mixer for 1 h at room temperature. Then the samples were washed 3 times with separation buffer and subjected to flow cytometry for detection. To eliminate the nonspecificity of dyeing and gating appropriate area, isotype control antibodies (ALL purchased from BD) were labeled simultaneously.

### 2.4. Effects of Tfh Cell-Derived Exosomes on B Cells

Lymphocytes in peripheral blood of patients in each group were separated by using a lymphocyte separation solution (TBD company). CD4+T cells in lymphocytes were firstly sorted through negative sorting by using CD4+T cell sorting Kit (BD). Then CD4+CXCR5+cells were sorted through positive sorting by magnetic beads coated with CXCR5 antibody (BD). CD4+CXCR5+cells were cultured in RPMI1640 (Hyclone) containing 10% exosome-free fetal bovine serum (Gibco). After 48 hours of culture, the exosomes in the supernatants were separated. Then, exosomes were added to the medium of lymphocytes that were depleted of Tfh cells, and cultivation was continued for 48 hours. SEB could promote T cell proliferation and secretion of many cytokine, so the lymphocytes (Tfh cell-) in each group were cultured in the cell culture medium with 1 mg/ml SEB. The cells were added with anti-CD3, anti-CD19, and anti-CD38 flow antibodies (ALL purchased from BD), incubated on the sample mixer for 20 min at room temperature. Then the samples were washed 3 times with PBS and the proportion of B cells (CD3-CD19+ cells) and CD38++ plasma cells (CD3-CD19-CD38+ cells) was measured by flow cytometry. Ig concentrations were measured by ELISA kits (ALL purchased from Huijia Biotechnology).

### 2.5. Statistical Analysis

Quantitative data was expressed as mean ± SD. SPSS 19.0 software was used to statistically process data. Two-sided Wilcoxon rank sum test was used to compare the clinical data. Spearman rank test correlation and paired t-test were used to compare experimental data. The significance level was set as P <0.05.

## 3. Results

### 3.1. General Information of the Patients

Our study population consisted of 42 patients after renal transplantation. After renal transplantation, all patients had similar immunosuppressive regimens with tacrolimus, mycophenolate mofetil, and prednisone acetate. There was no significant difference in the total dose of immunosuppressive agents. Baseline data were shown in Table 1 and Table S1. Our study included 28 patients with CRAD. In the CRAD group, 14 patients were diagnosed with AMR based on 2013 Banff Criteria (moderate or severe microvascular inflammation, positive serum DSA and positive C4d staining in the graft, exclusion of thrombotic microangiopathy), 14 patients were diagnosed with non-AMR (microvascular inflammation was not detected, negative serum DSA and negative C4d staining in the graft).

### 3.2. Characteristics of Exosomes

**Figure 1:**
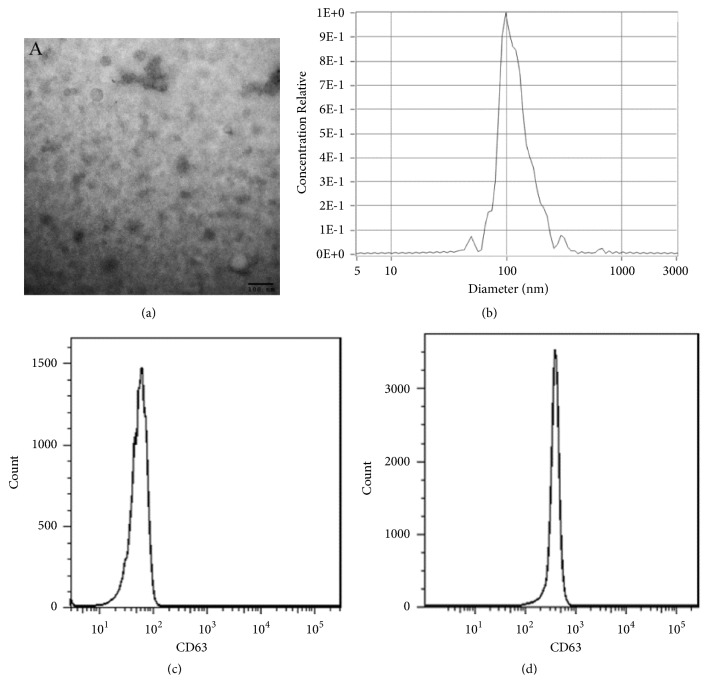
Characteristics of exosomes. (a, b) Transmission electron microscopy and nanoparticle tracking analysis showed the characteristic of exosomes; (c) flow cytometry results showed isotype control for CD63; (d) over 95% of exosomes expressed CD63.

The results of transmission electron microscopy showed that our isolated exosomes were either tea tray type or hemispherical with a concave side, with a diameter of 50-100 nm (Figure 1(a)); nanoparticle tracking analysis (NTA) results showed the diameter of exosomes (Figure 1(b)); we further used magnetic beads coated with CD63 antibody bound to exosomes; flow cytometry results showed that over 95% of exosomes expressed CD63 (Figures 1(c) and 1(d)).

### 3.3. Comparison of Exosomes and Their Subtypes

**Figure 2:**
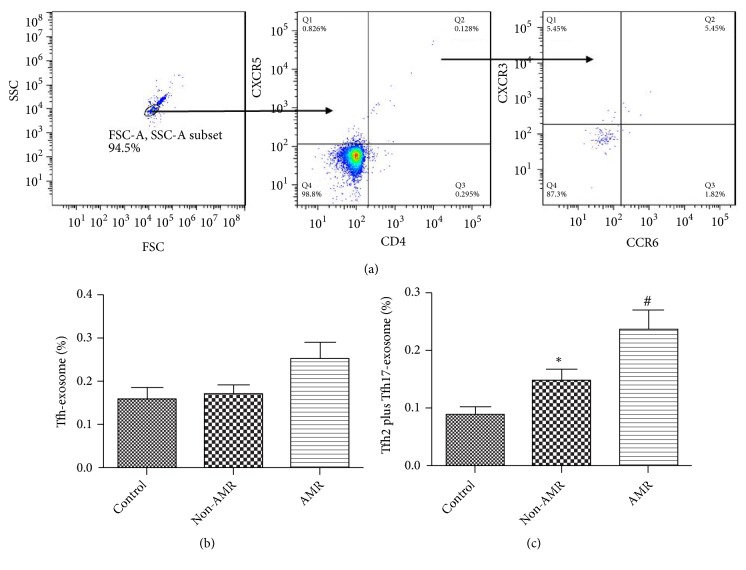
Comparison of CD4+CXCR5+exosomes and their subtypes. (a) CD4+CXCR5+exosomes and their subtypes were detected by flow cytometry; (b) there was no significant difference in the proportion of CD4+CXCR5+exosomes between AMR and non-AMR group; (c) the proportion of CD4+CXCR5+CXCR3-exosomes in the AMR group was significantly higher than that in non-AMR group. *∗*p<0.05 (n=14) versus non-AMR. Values are mean ± SD.

Flow cytometry results showed no significant difference in the percentage of CD4+CXCR5+exosomes in CRAD group and control group. In CRAD group, the proportion of CD4+CXCR5+CXCR3-exosomes was higher than that in control group. CRAD patients were further divided into AMR group and non-AMR group. The results showed that there was no significant difference in creatinine level, CD4+CXCR5+, and CD4+CXCR5+CXCR3+CCR6-exosomes between the two groups, while the proportion of CD4+CXCR5+CXCR3-exosomes in AMR group was significantly higher than that in non-AMR group (Figure 2).

### 3.4. HLA-G and CTLA-4 Expression in CD4+CXCR5+Exosomes

**Figure 3:**
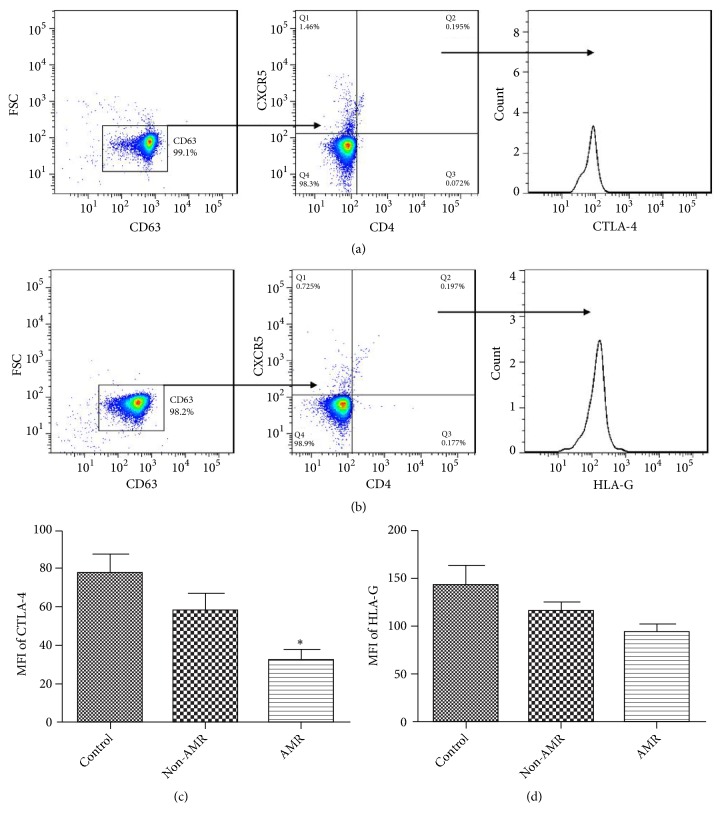
Comparison of HLA-G and CTLA-4 expression in CD4+CXCR5+exosomes. The expression of CTLA-4 (a) and HLA-G (b) on CD4+CXCR5+exosomes was detected by flow cytometry; (c) the expression of CTLA-4 on CD4+CXCR5+exosomes in AMR group was significantly lower than that in non-AMR group; (d) there was no significant difference in the expression of HLA-G between AMR and non-AMR group. *∗*p<0.05 (n=14) versus non-AMR. Values are mean ± SD.

Many papers reported HLA-G and CTLA-4 could induce immune tolerance of allografts and was considered as therapeutic target for future immunosuppressive drugs [14–17]. Our results showed HLA-G and CTLA-4 had no significant changes in the expression of CD4+CXCR5+exosomes in CRAD and control group. In CRAD patients, the expression of CTLA-4 on CD4+CXCR5+exosomes in the AMR group was significantly lower than that in the non-AMR group (Figure 3(c)), but there was no significant difference in the expression of HLA-G between two groups (Figure 3(d)).

### 3.5. Correlation between Exosomes and Clinical Data

There was no significant correlation between DSA and CD4+CXCR5+ exosomes, and there was no significant correlation between HLA-G expression on CD4+CXCR5+exosomes and DSA. CTLA-4 expression on CD4+CXCR5+exosomes was negatively correlated with DSA. We further analyzed the association of CD4+CXCR5+exosomes with DSA and found no significant correlation between the ratio of DSA and Tfh1 cell-derived exosomes. The proportion of CD4+CXCR5+CXCR3-exosomes was positively correlated with DSA. These results suggested that the decreased expression of CTLA-4 on CD4+CXCR5+exosomes and the increase in CD4+CXCR5+CXCR3-exosomes may be associated with the occurrence of AMR after renal transplantation.

### 3.6. CTLA-4 Expression on Tfh Cell-Derived Exosomes Was Reduced in Patients with AMR

**Figure 4:**
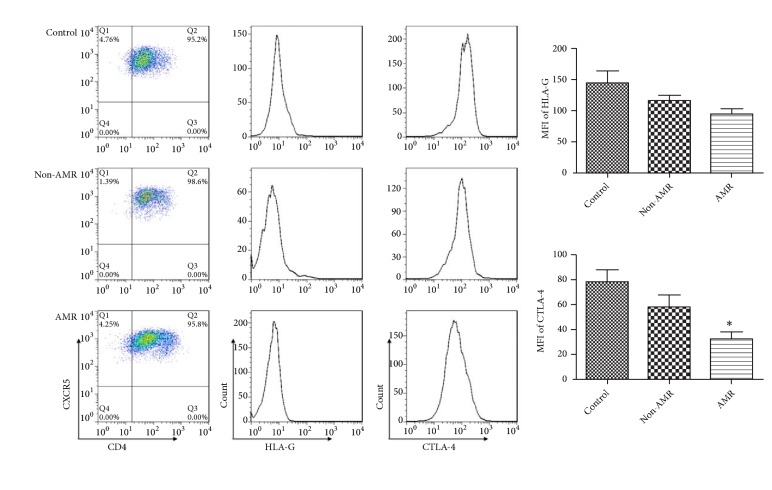
The expression of CTLA-4 on Tfh cell-derived exosomes was reduced in patients with AMR. Tfh cells of patients were separated and then cultured for 48 h. The exosomes in the supernatants were separated and their expression of CTLA-4 and HLA-G on Tfh cell-derived exosomes was detected by flow cytometry. The expression of CTLA-4 in Tfh cell-derived exosomes was significantly lower in AMR patients than that in non-AMR patients. However, there was no significant difference in the expression of HLA-G between two groups. *∗*p<0.05 (n=6) versus non-AMR. Values are mean ± SD.

We further separated CD4+CXCR5+cells from each group of patients. After 48 hours of culture, the exosomes in the supernatants were separated. Flow cytometry results showed no significant changes in the expression of HLA-G and CTLA-4 in Tfh cell-derived exosomes in CRAD and control group. CTLA-4 expression in Tfh cell-derived exosomes was significantly lower in AMR patients than that in non-AMR patients. However, there was no significant difference in the expression of HLA-G between two groups (Figure 4).

### 3.7. Tfh Cell-Derived Exosomes in AMR Patients Promote B Cell Proliferation and Differentiation

**Figure 5:**
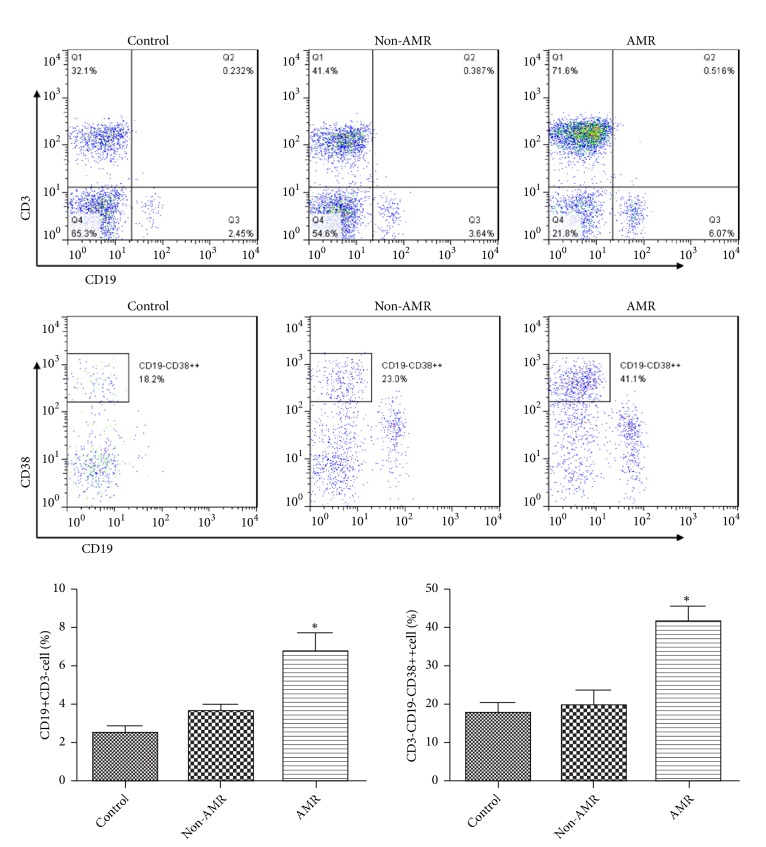
Tfh cell-derived exosomes in AMR patients promote B cell proliferation and differentiation. The proportion of CD3-CD19+B cells and CD3-CD19-CD38++ plasma cells was detected by flow cytometry. Tfh cell-derived exosomes in AMR group can further promote the proliferation and differentiation of B cells. *∗*p<0.05 (n=6) versus non-AMR. Values are mean ± SD.

To examine whether Tfh cell-derived exosomes can smoothly access into B cells, exosomes were stained using PKH67 and then added to the B cell culture medium (Figure S1). The results suggested that B cells can phagocytize Tfh cell-derived exosomes. Flow cytometry results showed that compared with the control group, the proportion of B cells and plasma cells in non-AMR group increased by 45.78% and 11.82%, respectively. However, these increases were not statistically significant. Tfh cell-derived exosomes in AMR group can further promote the proliferation and differentiation of B cells, and the proportions of B cells and plasma cells increased by 87.52% and 110.2%, respectively, relative to the non-AMR group (Figure 5). ELISA results showed that Tfh cell-derived exosomes in AMR patients could promote plasma cell production of IgG and IgA, but had no significant effect on IgM production (Figure S2).

## 4. Discussion

AMR is an important cause of late transplanted kidney function loss, and the current prevention and treatment effects are unsatisfactory. The production of DSA depends on the help of Tfh cells. Tfh2 and Tfh17 cells can promote B cell proliferation and differentiate into plasma cells through secreting large amounts of IL-21 [18, 19]. Exosomes are rich in biomarkers and have the function of delivering biomolecules and mediating cell-to-cell communication. In peripheral blood, CD4+CXCR5+cells can be defined as Tfh cells and most of CD4+CXCR5+exosomes in peripheral blood come from Tfh cells. It should be noted that some central memory CD4+T cells and Tfr cells can also release CD4+CXCR5+exosomes, but they are very few relative to Tfh cells. The proportion of IL-21-producing Tfh cell subsets (Tfh2 and Tfh17 cells) was significantly higher in the AMR group than that in non-AMR group, these increased cells may release more exosomes into peripheral blood [8]. Our study found that the proportion of Tfh2 and Tfh17 cell-derived exosomes in the peripheral blood of AMR patients was significantly higher than that of non-AMR patients, suggesting that IL-21 producing Tfh cells (Tfh2 and Tfh17) derived exosomes were closely related to AMR.

IL-21 plays a key role in the proliferation and differentiation of B cells. Tfh cell can be divided into three major cell subpopulations: CXCR3+CCR6-Tfh1 cells, with the characteristics of Th1 cells; CXCR3-CCR6-Tfh2 cells, with Th2 cell characteristics; and CXCR3-CCR6+ Tfh17 cells, with the characteristics of Th17 cells. Among them, only Tfh2 and Tfh17 cells can secrete IL-21 to induce proliferation and differentiation of primary B cells into plasma cells [20, 21]. Our study found that Tfh cell-derived exosomes promoted B cell proliferation and differentiation. Tfh cell-derived exosomes in AMR patients had a stronger effect on B cells than those of non-AMR patients. This may be due to the presence of more IL-21 in IL-21 producing Tfh cells (Tfh2 and Tfh17) derived exosomes. Detection of IL-21 producing Tfh cells derived exosomes can effectively predict the occurrence of AMR.

HLA-G plays an important role in exosomes-induced immune tolerance [22, 23]. However, our study found that HLA-G expression was not significantly different between AMR patients and non-AMR patients, indicating that the role of HLA-G in the occurrence and development of AMR was not very significant. CTLA-4 is a leukocyte differentiation antigen and a transmembrane receptor on T cells. It shares the B7 ligand with CD28. CTLA-4 has a significant therapeutic effect on transplant rejection and various autoimmune diseases [24–26]. Our study found that CTLA-4 expression on the surface of Tfh cell-derived exosomes was significantly reduced in AMR patients. CTLA-4 on exosomes directly interacted with the CD80 or CD86 molecule, further inhibited human T cell activation. Intracellular CTLA-4 can also inhibit the differentiation of Tfh cells, reduce the secretion of IL-21, and inhibit B cell proliferation and differentiation into plasma cells.

## 5. Conclusion

CD4+CXCR5+CXCR3-exosomes increased in AMR after renal transplantation; Tfh cell-derived exosomes promoted B cell proliferation and differentiation. Our research provides some theoretical support for AMR research.

## Figures and Tables

**Table 1:** The baseline and clinical characteristics of CRAD patients in renal transplantation.

	CRAD (n=28)	AMR (n=14)	Non-AMR (n=14)	*P *value
Age (yr)	41.9 ± 12.8	41.0 ± 10.8	42.9 ± 13.7	0.681
Male gender	17	9	8	0.421
BMI (Kg/m^2^)	22.9 ± 3.6	23.8 ± 3.5	22.0 ± 3.8	0.197
Time after transplantation	5.18 ± 1.73	5.57 ± 1.27	4.78 ± 1.99	0.533
White blood cell	7.05 ± 2.8	7.7 ± 3.3	6.4 ± 2.3	0.481
Lymphocyte	1.60 ± 0.8	1.63 ± 1.0	1.56 ± 0.7	0.886
Monocyte	0.54 ± 0.3	0.58 ± 0.3	0.50 ± 0.3	0.409
Urea nitrogen	18.8 ± 9.7	20.5 ± 11.0	17.1 ± 9.1	0.257
Creatinine (umol/L)	359.3 ± 212.4	405.6 ± 231.1	312.9 ± 202.7	0.227
Uric acid	452.2 ± 151.5	467.2 ± 145.3	437.2 ± 155.8	0.587
Total protein	66.1 ± 8.6	68.1 ± 10.2	64.1 ± 7.8	0.198
Triglyceride	1.86 ± 0.73	1.93 ± 0.72	1.79 ± 0.75	0.598
Total cholesterol	4.36 ± 1.26	4.15 ± 1.35	4.57 ± 1.23	0.357

## Data Availability

The data used to support the findings of this study are available from the corresponding author upon request.

## Supplementary Materials

Supplementary Materials Table S1: the baseline and clinical characteristics of recipients in renal transplantation. Figure S1: B cells phagocytized Tfh cell-derived exosomes. Figure S2: Tfh cell-derived exosomes in AMR patients promoted Ig production.
