# Supplementary material for: Follicular Helper T Cell Derived Exosomes Promote B Cell Proliferation and Differentiation in Antibody-Mediated Rejection after Renal Transplantation
Source: Biomed Res Int. 2019 May 15;2019:6387924. doi: 10.1155/2019/6387924 (PMC6541933; doi:10.1155/2019/6387924)

Follicular helper T cell derived exosomes promote B cell proliferation and differentiation in antibody-mediated rejection after renal transplantation.

Jintao Yang ^(a,b)^, Lili Bi ^b^, Xiuyun He ^b^, Zhen Wang ^b^, Yeyong Qian ^b^, Li Xiao ^b, †^, Bingyi Shi ^b, †^

a. Chinese PLA Medical School, Chinese PLA General Hospital, Beijing 100853, China

b. Beijing Key Laboratory of Immunology Regulatory and Organ Transplantation, Basic Research Lab of Organ Transplant Institute, The Eighth Medical Center Of Chinese PLA General Hospital, Beijing 100091, China

^†^ Corresponding author: Bingyi Shi, E-mail: shibingyi@medmail.com.cn;

Li Xiao, E-mail: xiaolilab309@163.com

Table S1. The baseline and clinical characteristics of recipients in renal transplantation

|  | Total (n=42) | CRAD (n=28) | Control (n=14) |
| --- | --- | --- | --- |
| Age (yr)  Male gender  BMI (Kg/m2)  Time after transplantation  White blood cell  Lymphocyte  Monocyte  Urea nitrogen  Creatinine (umol/L)  Uric acid  Total protein  Triglyceride  Total cholesterol | 43.07±12.3  24  23.3±3.8  4.64±1.69  7.23±2.5  1.58±0.71  0.55±0.28  14.4±7.3  263.3±159.3  411.1±96.7  66.8±9.4  1.82±0.81  4.28±1.16 | 41.9±12.8  17  22.9±3.6  4.86±1.73  7.05±2.8  1.60±0.8  0.54±0.3  18.8±9.7  359.3±212.4  452.2±151.5  66.1±8.6  1.86±0.73  4.36±1.26 | 45.4±11.6  7  24.1±4.2  4.2±1.61  7.6±1.9  1.53±0.64  0.56±0.26  5.6±1.1  71.3±12.6  329±72  68.3±11.3  1.75±0.95  4.13±0.93 |

Figure S1. Tfh cell-derived exosomes were stained using PKH67 and then added into the B cell culture medium. After culture for 24h, cell fluorescence intensity was observed by under fluorescence inverted microscope. The results suggested that B cells can phagocytize Tfh cell-derived exosomes.


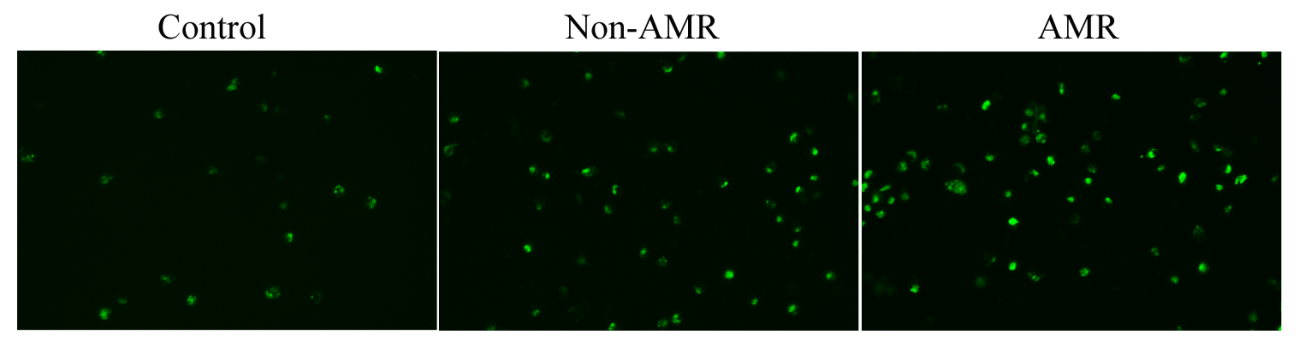


Figure S2. ELISA results showed that Tfh cell-derived exosomes in AMR patients could promote plasma cell production of IgG and IgA, but had no significant effect on IgM production.


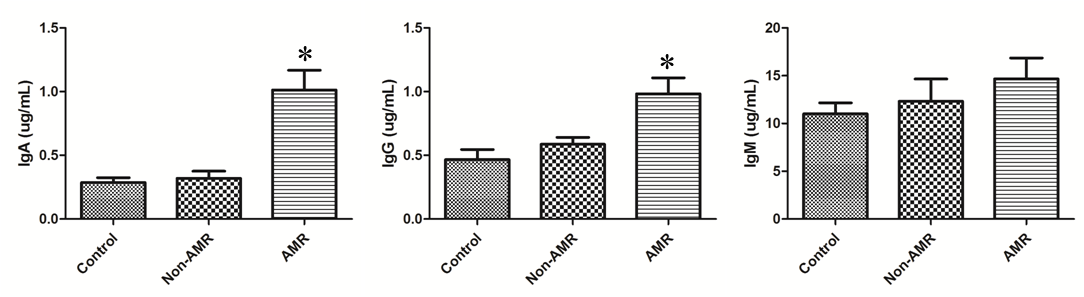

Supplement: Supplementary Materials — Table S1: the baseline and clinical characteristics of recipients in renal transplantation. Figure S1: B cells phagocytized Tfh cell-derived exosomes. Figure S2: Tfh cell-derived exosomes in AMR patients promoted Ig production. [file 6387924.f1.docx]
